# GPT2 mediates metabolic alterations in platinum-resistant ovarian cancer cells

**DOI:** 10.21203/rs.3.rs-6480518/v1

**Published:** 2025-05-09

**Authors:** Adriana Ponton-Almodovar, Mary Priyanka Udumula, Vrinda Khullar, Faraz Rashid, Ramandeep Rattan, Jamie J. Bernard, Sachi Horibata

**Affiliations:** Michigan State University; Henry Ford Health System; Michigan State University; Henry Ford Health System; Henry Ford Health System; Michigan State University; Michigan State University

**Keywords:** ovarian cancer, metabolism, GPT2, glutamine

## Abstract

Metabolic reprogramming is recognized as a hallmark of cancer frequently associated with drug resistance in ovarian cancer. This is problematic as ovarian cancer is one of the deadliest gynecologic cancers with platinum resistance contributing to poor survival. However, the mechanism by which ovarian cancer cell metabolism contributes to platinum resistance is not well understood. Herein, metabolic signatures were determined in platinum-resistant ovarian cancer cell lines compared to the more platinum-sensitive parental lines. Chemoresistant ovarian cancer cells showed increased oxidative phosphorylation (OXPHOS) compared to chemosensitive cells. This was associated with elevated levels of glutaminolysis and tricarboxylic acid (TCA)-related metabolites supporting their dependence on OXPHOS. Key enzymes involved in glutaminolysis, specifically, glutamic-pyruvic transaminase 2 *(GPT2),* were upregulated in chemoresistant compared to chemosensitive cells. Interestingly, high *GPT2* gene expression is associated with worse prognosis in ovarian cancer patients, adding translational relevance to the pre-clinical findings. GPT2 knockout in chemoresistant cells restored the metabolic phenotype to that of the sensitive cells and reversed drug resistance. These data suggest that GPT2 is a critical link between glutaminolysis, the TCA cycle, and OXPHOS and is a potential target to attenuate the increased metabolic activity associated with a chemoresistant phenotype.

## Introduction

Ovarian cancer is expected to be the second leading cause of death from gynecologic cancers in the United States in 2025^1^. This lethal disease is often diagnosed in an advanced or late stage, resulting in less effective treatment outcomes and increased morbidity and mortality^2^. The first-line chemotherapy consists of a combination of paclitaxel and platinum-based drugs, such as carboplatin or cisplatin^3,4^ While most patients initially respond to this chemotherapy combination, approximately 70–80% will develop chemoresistance^3,4^ Cisplatin and carboplatin directly target the DNA by binding to the purine nucleotides and creating DNA adducts^4^. This results in DNA damage and, ultimately, cell death via apoptosis^5,6^ Several mechanisms of platinum resistance have been established, including an increase in DNA damage repair mechanisms^7,8^ downregulation of the CTR1 influx pump^9,10^ and upregulation of ATPA/7B efflux pumps^11–13^. To date, none of these mechanisms have been amenable to pharmacological intervention demonstrating a continued need to understand how ovarian cancer cells grow in the presence of platinum-based drugs.

Cancer cells can adapt their metabolism to fulfill energy demands for proliferation, rapid growth, invasion, and drug resistance^14,15^. Glycolysis and oxidative phosphorylation (OXPHOS) are the two major metabolic pathways in which cells can generate energy in the form of ATP^16,17^ The ability to switch between glycolysis and glutamine-driven OXPHOS enables cancer cells to adapt and survive under stressed conditions^14,18^

Previously, we have shown that chemosensitive cancer cell lines (A2780 and PEO1) could not survive without glucose while their chemoresistant counterparts (C200 and PEO4) maintained their energy production under glucose deprivation conditions^19^. This potentially indicates metabolic reprogramming towards other carbon sources; however, usage of other potential sources is not yet well established in ovarian cancer. Altered glutamine metabolism in platinum resistant ovarian cancer cells have been reported to be driven by glutaminase (GLS)^20^. Herein, we characterized the glutaminolysis pathway as a carbon source in the ovarian cancer cells and expanded on the glutaminolysis-related enzymes to determine the metabolic alterations that occur in chemoresistant ovarian cancer cells that allow them to continue to proliferate in the presence of cisplatin. In this study, we identified glutamic-pyruvic transaminase 2 (GPT2) as a key player in glutamine-mediated chemoresistance.

## Results

### Cisplatin-resistant ovarian cancer cells are dependent on glutamine-driven oxidative phosphorylation

**Figure 1:**
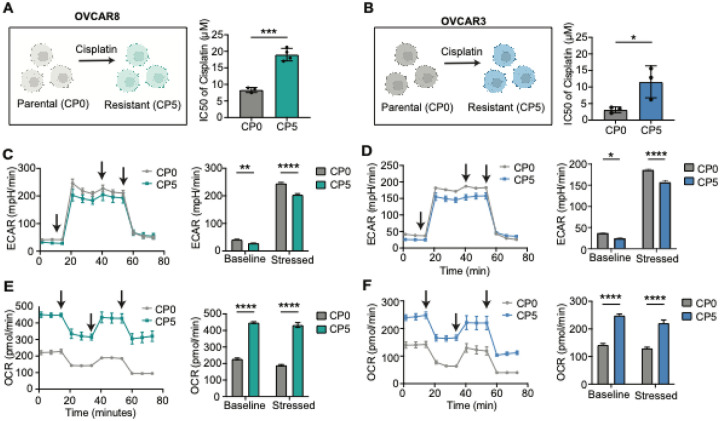
Cisplatin-resistant OVCAR8 and OVCAR3 cells show high oxidative phosphorylation. (A-B) Schematic depicting the development of cisplatin-resistant OVCAR8 (A) and OVCAR3 (B) cells. The IC50 levels were determined after treating the cells with cisplatin for 72h. CellTiter-Glo Viability Assay was used to test cell viability and survival. N=3 independent experiments, Unpaired t-test, *** p < 0.001, * p < 0.05. (C-D) Extracellular acidification rate (ECAR) of OVCAR8-CP0, OVCAR8-CP5, OVCAR3-CP0, and OVCAR3-CP5 cells. Arrows indicate the time of injection. The bar graph represents the baseline and stressed levels for ECAR. Two-Way ANOVA, * p < 0.05, ** p < 0.01, **** p < 0.0001. (E-F) Oxygen consumption rate (OCR) of OVCAR8-CP0, OVCAR8-CP5, OVCAR3-CP0, and OVCAR3-CP5 cells. Arrows indicate the time of injection. The bar graph represents the baseline and stressed levels for OCR. Two-Way ANOVA, **** p <0.0001.

The Seahorse metabolic assay was conducted to assess metabolic alterations in parental OVCAR8 and OVCAR3 ovarian cancer cell lines (CP0) and their cisplatin-resistant counterparts (CP5) (Figs. 1A–B). Evaluation of glycolysis, as measured by extracellular acidification rate (ECAR), revealed low baseline levels of glycolytic activity in OVCAR8-CP5 (Fig. 1C) and OVCAR3-CP5 (Fig. 1D) compared to their parental cells. Under stressed conditions, the glycolytic activity increased in both parental and cisplatin-resistant cells but was higher in the parental cells (Figs. 1C–D). On the other hand, evaluation of OXPHOS, as measured by oxygen consumption rate (OCR) showed significantly increased levels of OXPHOS activity in cisplatin-resistant OVCAR8-CP5 (Fig. 1E) and OVCAR3-CP5 (Fig. 1F) compared to their parental cells under basal and stressed conditions.

In parallel, we conducted untargeted metabolomics on the ovarian cancer cells to discern the metabolomic profile of cisplatin-resistant ovarian cancer cells compared to their parental cells. A total of 105 metabolites were evaluated and their differential abundance of metabolites were compared between the cisplatin-resistant and parental cells (Supplementary Fig. 1). We detected no significant changes in arginine, aspartic acid, histidine, lysine, glutamic acid, alanine, threonine, asparagine (Supplementary Fig. 1). However, we observed significantly lower levels of serine, methionine, isoleucine, valine, phenylalanine and tyrosine in cisplatin-resistant cells compared to their parental cells (Supplementary Fig. 1). Among all amino acids, glutamine levels were the highest in the cisplatin-resistant ovarian cancer cell models compared to their parental cells (Supplementary Fig. 1).

### Cisplatin-resistant cells show increased in metabolites involved in glutaminolysis and TCA cycle

**Figure 2:**
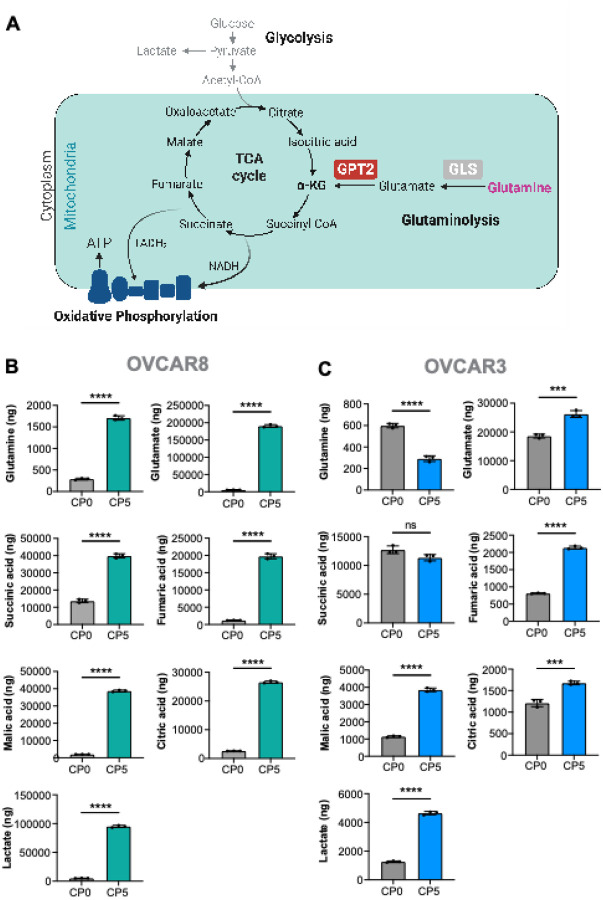
Cisplatin-resistant cells exhibit high levels of metabolites involved in glutaminolysis and the TCA cycle compared to their parental cells. (A) Schematic illustration of glycolysis, the TCA cycle, glutaminolysis, and oxidative phosphorylation. (B-C) Targeted analysis of the glutaminolysis metabolites glutamine and glutamate, the TCA cycle intermediates citric acid, succinic acid, fumaric acid, and malic acid, as well as lactate using LC/MS was performed in cisplatin-resistant OVCAR8 (B) and OVCAR3 (C) models. N=3 individual experiments, Unpaired t-test, ns = not significant, *** p < 0.001, **** p < 0.0001.

A targeted analysis of metabolites involved in pathways affected by glutamine, such as glutaminolysis and TCA cycle was conducted (Fig. 2A). OVCAR8-CP5 demonstrated significantly elevated levels of glutamine, glutamate, citric acid, succinic acid, fumaric acid, malic acid, and lactate (Fig. 2B). Similarly, OVCAR3-CP5 demonstrated significantly elevated levels of glutamate, citric acid, fumaric acid, malic acid, and lactate (Fig. 2C). However, succinic acid level was not statistically different between the parental and OVCAR3-CP5 cells. Interestingly, we observed low levels of glutamine in OVCAR3-CP5 compared to their parental cells.

**Figure 3:**
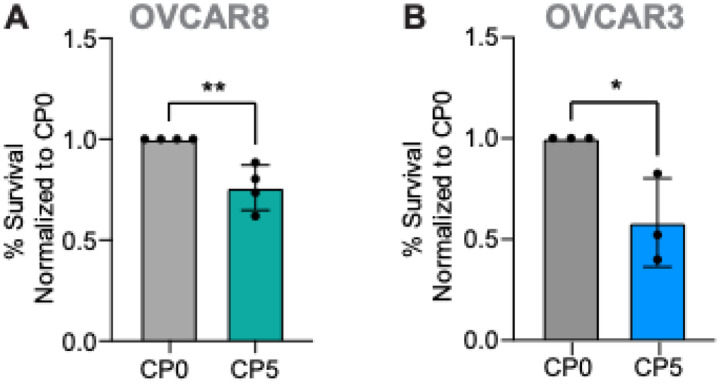
Cisplatin-resistant OVCAR8 and OVCAR3 cells are sensitive to glutamine deprivation. (A) Normalized % survival of cisplatin-resistant OVCAR8 (CP5) cells and parental cells (CP0) grown in glutamine and FBS deprived media containing 5% FBS. N=4 individual experiments, student’s test, ** p < 0.01. (B) Normalized % survival of cisplatin-resistant OVCAR3 (CP5) cells and parental cells (CP0) grown in glutamine and FBS deprived media containing 5% FBS. N=3 individual experiments, student’s t-test, * p < 0.05.

In order to determine the dependency of cisplatin-resistant cells to glutamine, we assessed the effects of glutamine deprivation on cellular viability using glutamine and FBS deprived media. We found that glutamine deprivation significantly killed cisplatin-resistant cells compared to the parental cells, indicating the dependency of cisplatin-resistant cells to glutamine (Fig. 3A–B).

### GPT2 potentially drives glutaminolysis in platinum-resistant ovarian cancer cells

**Figure 4:**
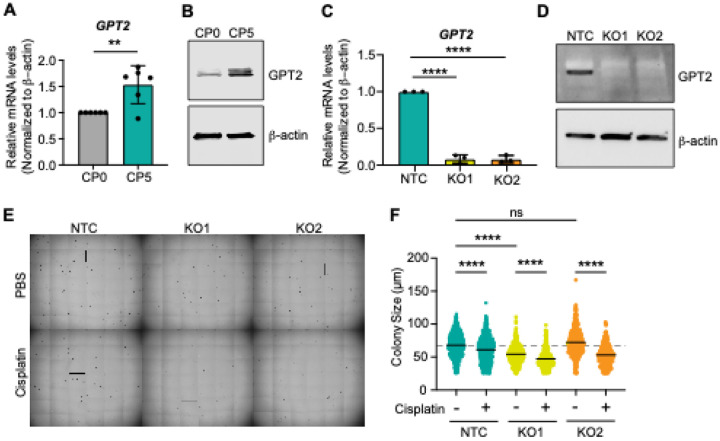
GPT2 is upregulated in cisplatin-resistant cells compared to the parental cells and complete GPT2 knockout clones show reduced colony size and increased sensitivity to cisplatin treatment in an anchorage-independent growth assay. (A) qPCR analysis showing the relative mRNA expression levels of *GPT2*for cisplatin-resistant OVCAR8 (CP5) cells compared to the parental cells (CP0). N=6 individual experiments, student’s t-test; ** p < 0.01. (B) Western blot images of GPT2 protein expression level in OVCAR8CP0 and OVCAR8CP5 cells. b-actin is used as a reference control. (C) qPCR of CRISPR-knockout of GPT2 (KO) in resistant cells. Two clonal knockout cells were generated (KO1 and KO2). (D) Western blot images of CRISPR-knock out of GPT2 in resistant cells. b-actin was used as a reference control. (E) Representative wide-field images of non-targeting control (NTC) and two GPT2-KO cells grown in soft agar in the presence or absence of 1 mM cisplatin. (F) Colony size of NTC, KO1, and KO2 cells using soft agar assays. N=3 individual experiments, Kruskal-Wallis non-parametric test, ns = not significant, **** p < 0.0001.

Next, a comprehensive qRT-PCR analysis was performed on our cisplatin-resistant model to identify a metabolic target involved in cisplatin resistance. We assessed the key enzymes involved in glutaminolysis: *GPT2, GLS,* and *GOT2* (Figs. 4A–B, Supplementary Figs. 2A-C) and *CS, IDH2, DLST, SUCLG2, SDHC, FH,* and *MDH2* for genes of enzymes involved in TCA cycle (Supplementary Fig. 3). From these, we identified *GPT2* mRNA and GPT2 protein to be upregulated in cisplatin-resistant OVCAR8 and OVCAR3 cells compared to their parental cells (Figs. 4A–B, Supplementary Fig. 2A). Thus, we identified GPT2 as a potential modulator of glutaminolysis towards the TCA cycle and OXPHOS in cisplatin-resistant cells.

To investigate whether GPT2 directly confers cisplatin resistance, we used CRISPR/Cas9 to knock out GPT2 in OVCAR8CP5 resistant cells (Figs. 4C–D) as well as generated their appropriate CRISPR controls (non-targeting control, NTC). Experiments with two clonal GPT2 knock-out (GPT2-KO1 and -KO2) cell lines showed that GPT2 knockout increased sensitivity of the GPT2 knock-out cells to cisplatin treatment and reduces colony growth (Figs. 4E–F).

### GPT2 knockout inhibits oxidative phosphorylation (OXPHOS)

**Figure 5:**
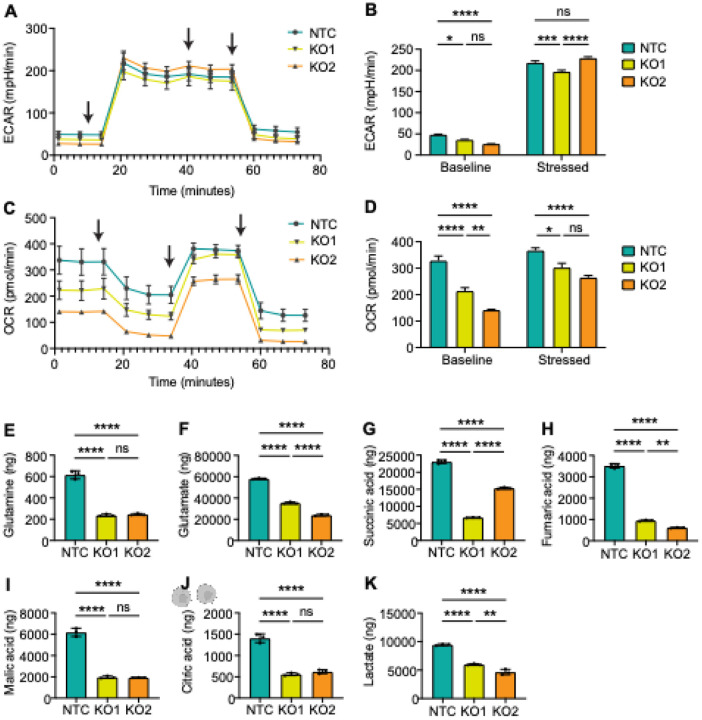
Complete GPT2 knockout in cisplatin-resistant cells have a similar metabolite signature to the less resistant parental cells. (A) Extracellular acidification rate (ECAR) of OVCAR8-CP5 CRISPR non-targeting control (NTC) and OVCAR8-CP5 GPT2 knockout (KO1 and KO2) cells. Arrows indicate the time of injection. (B) The bar graph represents the baseline and stressed levels for ECAR. Two-Way ANOVA, ns = not significant, * p < 0.05, *** p < 0.001, **** p < 0.0001. (C) Oxygen consumption rate (OCR) of OVCAR8- NTC, -KO1, and -KO2. Arrows indicate the time of injection. (D) The bar graph represents the baseline and stressed levels for OCR. Two-Way ANOVA, ns = not significant, * p < 0.05, ** p < 0.01, **** p < 0.0001. (E-K) Targeted analysis of glutamine (E), glutamate (F), succinic acid (G), fumaric acid (H), malic acid (I), citric acid (J), and lactate (K). N=3 individual experiments, one way ANOVA, ns = not significant, ** p < 0.01, **** p < 0.0001.

Lastly, the Seahorse metabolic assay was conducted to address whether increased cisplatin sensitivity of GPT2 knock out cells are linked to OXPHOS. Glycolysis was used as a control. We observed a slight decrease in glycolysis/ECAR baseline levels in GPT2 knock-out clones and variable levels under stressed conditions (Figs. 5A–B). However, we observed a significant decrease in OXPHOS/OCR baseline levels in GPT2-KO clones, as well as under stress conditions compared to cisplatin-resistant control (Figs. 5C–D). Targeted analysis of TCA cycle and glutaminolysis-related metabolites demonstrated significantly decreased levels of glutamine, glutamate, citric acid, succinic acid, fumaric acid and malic acid (Figs. 5E–K). Thus, GPT2 mediates metabolic alteration, specifically OXPHOS, in cisplatin resistant ovarian cancer cells.

## Discussion

Since the identification of metabolic reprogramming as a hallmark of cancers, the role of glutamine metabolism in tumor growth, metastasis, and chemoresistance has been gaining attention in ovarian cancer field^20–23^. Glutamine is a non-essential amino acid that supports anaplerosis^24,25^. The latter is a metabolic process that allows the replenishment of glutamine-derived intermediates to sustain the rapid proliferation of cancer cells and to adapt to metabolic demands^18^. One study demonstrated that highly invasive ovarian cancer cells are dependent on glutamine for proliferation and tumor growth^21^. In their invasive model, glutamine was found to play a key role in supporting mitochondrial function. Specifically, it facilitates an increase in glutathione production–reducing reactive oxygen species. Thereby protecting the mitochondrial integrity and promoting the survival and proliferation of ovarian cancer cells^21^. Furthermore, a recent study focused on cisplatin-resistant ovarian cancer cells demonstrated that resistant cells rely on glutamine metabolism^22^, highlighting the metabolic adaptations that underlie resistance in ovarian cancer by exploiting glutamine utilization for survival. However, the exact players of this metabolic adaptation for cisplatin-resistance have not been fully characterized.

Herein, we characterized the metabolic profile of cisplatin-resistant high grade serous ovarian cancer (HGSOC) cell lines by assessing glycolysis and OXPHOS activity. Our results demonstrated that the cisplatin-resistant ovarian cancer cells (OVCAR3-CP5 and OVCAR8-CP5) predominantly utilized OXPHOS, suggesting an increase in mitochondrial activity. These observations align with our previous findings as we established that chemoresistant ovarian cancer cells depend on OXPHOS and exhibit enhanced mitochondrial function^19,23^. Interestingly, this reliance on OXPHOS may imply enhanced TCA cycle activity. For instance, the TCA cycle is central for producing metabolites involved in nucleotide biosynthesis (e.g., oxaloacetate) and the electron carriers NADH and FADH_2_^26,27^ which are necessary for redox reactions to occur during OXPHOS for ATP production. Recently, we demonstrated that the inhibition of the TCA cycle utilizing the inhibitor CP1–613, which targets TCA cycle enzymes pyruvate dehydrogenase (PDHA1) and 2-oxoglutarate dehydrogenase (OGDH), significantly decreased the growth of xenografts derived from resistant ovarian cancer cells^23^. This finding reveals the critical role of TCA cycle activity in the survival of chemoresistant ovarian cancer. In our current study, we observed that cisplatin-resistant ovarian cancer cells show enhanced glutaminolysis-related metabolites, which were correlated with increased levels of TCA cycle intermediates. Notably, we also observed significantly increased lactate in the cisplatin-resistant cells compared to the cisplatin-sensitive counterparts. The increase in lactate levels suggests metabolic reprogramming as described by the Warburg effect^28^ Altogether, these results strongly indicate that HGSOC platinum-resistant cells are capable of rewiring their metabolism towards glutaminolysis to enhance mitochondrial activity.

To identify a potential target that mediates mitochondrial activity, we evaluated genes involved in glutaminolysis and the TCA cycle. Glutaminolysis begins with the conversion of intracellular glutamine into glutamate, catalyzed by the enzyme glutaminase (GLS)^18,24,27^. Notably, a previous study demonstrated that platinum-resistant ovarian cancer cells exhibit high levels of GLS expression^20^. This study emphasizes the role of glutaminolysis in platinum resistance and establishes that this is driven by high GLS expression^20^. In our study, we observed that GLS is overexpressed in the OVCAR8CP5 cell line while remaining comparatively low in the OVCAR3CP5 cell line. Nonetheless, our targeted metabolomics revealed that both cell lines exhibit significantly increased glutamate levels, thereby suggesting the active enzymatic function of GLS. Glutamate is a critical metabolite in fueling the TCA cycle, as it can be converted into α-ketoglutarate (or 2-oxoglutarate) through the activity of glutamate dehydrogenase 1 (GLUD1) or the mitochondrial aminotransferases such as glutamic-oxaloacetic transaminase 2 (GOT2) and glutamic-pyruvic transaminase 2 (GPT2)^18,27^

GPT2 is an important regulator of glutaminolysis. GPT2 is a mitochondrial enzyme that catalyzes the reversible transamination of glutamate and pyruvate, resulting in the production of α-ketoglutarate and alanine^29^. This enzyme aids in anaplerosis, maintaining the functionality of the TCA cycle activity in cancer cells^30^. Moreover, GPT2 has been implicated in the progression and tumorigenesis of breast cancer^31,32^ colorectal cancer^33^, and glioblastoma^34^. In these studies, GPT2 mediated a metabolic shift that enhances the production of α-ketoglutarate and facilitates ATP production for cell survival and growth^30^. To our knowledge, GPT2 has not been studied in the context of ovarian cancer. Our comprehensive gene expression analysis revealed a significant upregulation of *GPT2* in both OVCAR8 and OVCAR3 platinum-resistant cell lines. This finding was further corroborated by the observed elevated GPT2 protein levels in these cell lines. Herein, we identified GPT2 as the potential regulator of glutaminolysis, which supports the TCA cycle and OXPHOS for ATP production, ultimately leading to platinum resistance.

In order to investigate the role of GPT2 in the context of cisplatin resistance, we conducted knockout studies. The complete knockout of *GPT2* revealed its potential involvement in tumorigenesis and platinum-resistance mechanism. Our experiments using soft agar demonstrated a significant reduction in the size of colonies formed by the GPT2 knockout clones, both in conditions where the clones were treated with cisplatin and untreated. This finding showed that GPT2 knock-out prevents the ability of these cells to proliferate and form colonies and instead, increased sensitization to cisplatin treatment. Subsequently, GPT2 knockout disrupts glutaminolysis and TCA cycle activity as demonstrated by low OXPHOS activity and decreased TCA cycle intermediates. The lower baseline OXPHOS correlates with a metabolic shift, which more closely resembles cisplatin-sensitive ovarian cancer cells. This suggests that GPT2 knockout may reverse the cisplatin resistance phenotype. Furthermore, the decreased levels of TCA cycle metabolites reveal metabolic reprogramming that could enhance sensitivity to cisplatin-based chemotherapy as observed in the soft agar studies. Collectively, these results demonstrated that knocking out GPT2 leads to significant metabolic rewiring in cisplatin-resistant ovarian cancer cells, ultimately favoring resensitization to cisplatin treatment.

## Conclusion

Altogether, our results show that platinum-resistant high-grade serous ovarian cancer cell lines depend on OXPHOS. Our platinum-resistant models showed elevated glutaminolysis and TCA cycle-related metabolites, which are associated with the upregulation of the glutaminolysis-related enzyme GPT2. We demonstrated through our knockout studies that GPT2-driven glutaminolysis maintains the TCA cycle and OXPHOS activity, allowing the ovarian cancer cells to become resistant to platinum-based chemotherapy.

## Methods

### Cell Culture

The ovarian cancer cell lines OVCAR8 and OVCAR3 are a generous gift from Dr. Michael Gottesman (National Cancer Institute, National Institutes of Health). Cells were cultured in RPMI-1640 (Gibco; Cat# 11875–093) supplemented with 10% fetal bovine serum (FBS, Gibco; Cat# A52567–01) and 1% penicillin and streptomycin (Gibco; Cat# 15140163) at 37°C incubator with 5% CO_2_ supply. Parental cell lines OVCAR8 and OVCAR3 (also referred to as OVCAR8-CP0 and OVCAR3-CP0, respectively) were grown without cisplatin (CP). The resistant cell lines, OVCAR8-CP5 and OVCAR3-CP5, were generated as described previously^5^ and grown in medium supplemented with 5 μM cis-Diamineplatinum (II) dichloride (cisplatin; Sigma-Aldrich; Cat# P4394). Cisplatin is dissolved in PBS in all the experiments, as DMSO has been shown to interfere with cisplatin activity^35^. All cell lines were authenticated by short tandem repeat (STR) analysis using the American Type Culture Collection (ATCC) FTA Sample Collection Kit for Human Cell Authentication Service (ATCC, Cat# 135-XV). All cells were tested for mycoplasma using the MycoAlert Plus Mycoplasma Detection Kit (Lonza; Cat# LT07–118).

### Quantitative PCR analysis

OVCAR8-CPO, OVCAR8-CP5, OVCAR3-CP0, and OVCAR3-CP5 were lysed, and RNA was isolated using RNeasy Mini Kit (Qiagen Cat# 74104). A total of 1 μg of RNA was used to synthesize cDNA using the High-Capacity cDNA Reverse Transcription Kit (ThermoFisher, Cat# 4368814) with RNase Inhibitor (ThermoFisher, Cat# N8080119). Diluted cDNA samples were analyzed by real-time PCR using *Power* SYBR^™^ Green PCR Master Mix (ThermoFisher, Cat# 4367659) on Bio-Rad C1000 Touch Thermal Cycler CFX96 Real-Time System. Amplification data was analyzed using the Bio-Rad CFX Manager (version 3.1.1517.0823). The primary sequences are shown in Table 1.

### CRISPR/Cas9 Transfection

OVCAR8-CP5 were co-transfected using the GPT2 CRISPR/Cas9 KO Plasmid (h) (Santa Cruz Biotech, Cat# sc-403739) and GPT2 HDR Plasmid (h) (Santa Cruz Biotech, Cat# sc-403739-HDR) to knockout GPT2 following protocol provided by the company. Control CRISPR/Cas9 Plasmid (Santa Cruz Biotech, Cat# sc-418922) was used to create non-targeting control (NTC) clones. Successful transfected clones were selected using puromycin dihydrochloride (Santa Cruz Biotech, Cat# 108071). Clones were selected using the NanoCellect WOLF Cell Sorter of the Precision Health Program at Michigan State University. Clones were validated using quantitative PCR (validation at the RNA level) and western blotting (validation at the protein level).

### Western Blotting

OVCAR8-CP0, OVCAR8-CP5, OVCAR3-CP0, OVCAR3-CP5, OVCAR8-CP5 NTC, and OVCAR8-CP5 GPT2-KO cells were lysed using 1X RIPA buffer supplemented with protease and phosphatase inhibitors (Roche, Cat# 11836170001). Total protein concentration was quantified using the Pierce^™^ BCA Protein Assay Kits (ThermoFisher, Cat# 23227). Protein was separated using sodium dodecyl-sulfate polyacrylamide gel electrophoresis (SDS-PAGE), followed by immunoblotting. The following primary antibodies were used to evaluate protein expression: GPT2 (Santa Cruz Biotech, Cat# sc-398383); β-actin (Cell Signaling, Cat# 3700S), GAPDH (Cell Signaling, #Cat 2118S).

### Soft Agar Assay

OVCAR8-CP5 NTC and OVCAR8-CP5 GPT2-KO clones (500 cells per well) were seeded in a 0.3% agar layer on top of a 0.5% layer of agar supplement with complete RPMI media. PBS (control) and 1.0 μM of cisplatin (experimental condition) were added to the layer containing the cells. After 14 days, the colonies formed were fixed using 70% ethanol for 30 min. The ethanol was removed, and the colonies were fixed with 200 μL of 0.01% crystal violet. Colonies quantification and size measurements were analyzed using the BioTek Cytation 3 Cell Imager (Gen5 3.04 software; BioTek Instruments, Inc. Winooski, VT) in the Assay Development and Drug Repurposing Core (ADDRC) at Michigan State University.

### Seahorse Metabolic Assay

Seahorse analysis evaluates cellular metabolic processes such as glycolysis, as measured by extracellular acidification rate (ECAR), and oxidative phosphorylation (OXPHOS), as measured by oxygen consumption rate (OCR). OVCAR8-CP0, OVCAR8-CP5, OVCAR3-CP0, OVCAR3-CP5, OVCAR8-CP5 NTC, and OVCAR8-CP5 GPT2-KO cells were plated at a density of 6 × 10^4^ cells/well in cell-Tak coated XFe 96 cell plates. The OCR and ECAR rates were measured using an XFe 96 seahorse analyzer (Agilent Seahorse XFe96 Analyzers). For measuring OCR, cells were incubated in an XF base medium supplemented with 2 mmol/L glutamine, 10 mmol/L glucose, and 1 mmol/L pyruvate. Various metabolic inhibitors were injected to observe the effect of the oxidative phosphorylation and extracellular acidification rate. OCR measurements were recorded with port injections of (1) oligomycin (1 μmol), (2) carbonyl cyanide-p-trifluoromethoxyphenylhydrazone (FCCP) (0.5 μmol), and a combination of (3) rotenone-antimycin at 1 μmol. ECAR, an indicator of aerobic glycolysis, was measured by incubating cells in an XFe base medium supplemented with 2 mmol/L glutamine. ECAR measurements were recorded after injecting with (1) glucose (10 mM), followed by (2) oligomycin (2 μM), and (3) 2-deoxyglucose(2-DG) (100 mM)^36^

### Untargeted metabolomics

OVCAR8CPO, OVCAR8CP5, OVCAR3CP0, and OVCAR3CP5 cells were plated at a density of 2.5 × 10^5^ cells/well in a 6-well plate. Cells were washed with ice-cold 150 mM ammonium acetate (pH 7.3). Cold 80% methanol containing 13C,15N-labeled amino acids (Sigma Aldrich, 767964) and 5 mg/mL of ribitol (adonitol) as internal standard was added and incubated for 30 min at −80°C. Cells were scraped, and contents were transferred to a chilled microfuge tube. Contents were centrifuged at top speed for 10 min at 4°C. The protein pellet was quantified using the Pierce^™^ BCA Protein Assay kit (ThermoFisher) and utilized for normalization of metabolite data. The supernatant was evaporated using a speed-vac and utilized for mass spectrometry analysis.

Dried samples were reconstituted in 90:10 acetonitrile/water and analyzed using a Thermo Q-Exactive mass spectrometer interfaced with a Thermo Vanquish UHPLC. 5 mL of each sample was injected onto a HILIC column (Waters Acquity UPLC BEH-Amide, 1.7 μm, 2.1×100 mm) at 40°C. A 0.3 mL/min flow rate was used for a gradient separation that started at 100% mobile phase B (90:10 acetonitrile/water containing 20 mM ammonium acetate and 0.1% ammonium hydroxide) and 0% mobile phase A (90:10 water/acetonitrile containing 20 mM ammonium acetate and 0.1% ammonium hydroxide) and was held for 0.5 min. Mobile phase A was ramped to 50% at 8 min and then to 80% at 10 min, held at 80% until 11 min and then returned to 100% B at 11.1 min until 15 min.

A data-dependent MS/MS method was used with electrospray ionization in either negative mode and capillary voltage of 2.5 kV or positive ion mode with a capillary voltage of 3.5kV. The transfer capillary temperature was set at 256°C, sheath gas at 48, auxiliary gas at 11, probe heater at 413°C, and S-lens RF level at 50. Survey scans were acquired at 35,000 resolution, automatic gain control (AGC) target of 3E6, maximum inject time 100 ms, and m/z range of 70–1,050. The top 5 ions were selected for MS/MS with a resolution setting of 17,500, AGC target of 1E5, maximum inject time 50 ms, isolation window of 1.0, dynamic exclusion setting of 3s, and stepped normalized collision energy settings of 10, 30, and 60. MS/MS was performed the Mass Spectrometry and Metabolomics Core at Michigan State University.

### TCA metabolites LC-MS method for extraction and quantification

As per experimental design, all the metabolites were extracted using 80:20 acetonitrile and water mixture. TCA standards were reconstituted in (water and acetonitrile, 20:80) to prepare 10 mg/ml stock solutions. Linear standards curve at the range of 5–1000 ng/ml of each analyte (citric acid, aconitase, iso-citric acid, *α*-ketoglutaric acid, itaconate, α-ketoglutarate, succinic acid, fumarate, and malic acid) was produced from stock solutions in the relevant matrix. Each standard (100 *μ*l) was mixed to obtain a 7-point standard calibration curve within a range of 5–1000 ng/ml. Each internal standard (10 *μ*g/ml) (isotope-labelled TCA mixtures standards mix Sets 1 and 2 A) was spiked into all samples as an internal standard (final concentration) to allow quantification based on the ratio of the internal standard to each intermediate peak. Approximately, 3 million cells were rapidly rinsed by 2 mL of 37°C deionized water to the cell surface. Cells were collected in 2-ml tubes after washing, followed by adding 500 μL of extraction solution (acetonitrile and water [80:20]) and stored at −80°C for further processing. 10 μL of each isotope-labeled TCA mixtures standards mix (Sets 1 and 2 A) was added as an internal standard followed by vortex to mix. Three cycles of extraction were carried out by vertexing for 1 min followed by sonicating for 1 min (each). After sonication, samples were kept at −20°C for protein precipitation, followed by centrifugation at 15,000 rpm for 20 min at 4°C. Supernatants were loaded into pre-conditioned Phenomenex Strata XL-100 60 mg/3 ml cartridges (Torrance, CA) and passed through it using positive pressure manifold (Agilent Technologies). Flow through was collected subsequently and 100 μL of filtrate was mixed with 100 μL of water for the LC-MS/MS system. The TCA cycle and its intermediate metabolites were identified and quantified based on retention time and m/z match to injections of authentic standards, retention time and accuracy bases and were quantified using internal standard area for the respective metabolites. Phenomenex Luna-NH2, 2.0 × 150 mm, 3 μm column was used to achieve an optimal separation of all TCA intermediates. The flow rate of 0.25 ml/min was used with a mobile phase A (10 mM NH_4_OAc buffer at pH 9.8) and mobile phase B (acetonitrile). Column temperature was optimized at 25°C for the best chromatographic separation. The metabolites were eluted from the column at a flow rate of 0.25 ml/min using a gradient system (mobile phase B): 80% (0.01–0.5 min), 80→20% (0.5–18.0 min), 20% (18.0–19.5 min), 20 → 80% (19.5–22 min), and re-equilibration (22–25 min). TCA cycle intermediates were monitored using negative MRM (multi reaction monitoring) polarity. Identification was achieved based on retention time and MS/MS ion conformation. Seven calibration standards ranging from 5 to 1000 ng/ml were subjected to the full extraction procedure 3 times before analysis. The mean correlation coefficients of each metabolite were linear r2 > 99 and were obtained (*n=* 3) from 5 to 1000 ng/ml. The calibration curve, prepared in a control matrix, was constructed using peak area ratios of the calibration samples by applying a one/concentration weighting (1/x) linear regression model. All quality control sample concentrations were then calculated from their PARs against the calibration curve. The parameters for triple quadrupole detector mass spectrometry equipped with an electrospray ionization probe: Capillary, voltage, 3.5 kV for negative mode: Source temperature 120°C: Desolvation temp: 450°C; Cone gas flow: 150 L/h: Desolvation gas flow:1000 L/Hr Collision gas flow: 0.25 mL/min and nebulizer gas flow: 7 Bar^23,37^

### Statistical Analysis

All statistical analyses and statistical significance were determined using GraphPad Prism 10. Appropriate statistical tests are indicated in the figure legends.

## Figures and Tables

**Table 1:** Primer sequences were utilized for gene expression analysis using quantitative PCR.

Gene Name	Symbol	Left Primer	Right Primer
Citrate synthase	*CS*	AGAAACTGCTACCCAAGGCT	GGGGTGTAGATTGGTGGGAA
Aconitase 2	*ACO2*	ATGAAGATATGGGGCGCTCA	ATGTCCTTCCTGTCCCACTG
Isocitrate dehydrogenase 2	*IDH2*	GGCTCAGGTCCTCAAGTCTT	AGCCTCAATCGTCTTCCCAT
Oxoglutarate (α-ketoglutarate) dehydrogenase	*OGDH*	TTTGGTCTAGAAGGCTGCGA	TCTGTTCCAGCTCCTTCCTG
Dihydrolipoamide S-succinyltransferase	*DLST*	GCAAATGGCGTGATTGAAGC	ATGCTCAGTCCCAATCCCAA
Succinate-CoA ligase GDP-forming subunit β	*SUCLG2*	CCTGCTTTGTGAATGGTGCT	GCACAGTTGACGATACCACC
Succinate dehydrogenase complex subunit C	*SDHC*	CCTACTCTCGGCCTAGAAGC	GTCCTTTAAGCCAGCAACCC
Fumarate hydratase	*FH*	CCACTTACTCTTGGGCAGGA	GGAGCAGTGACAAAAGGCAA
Malate dehydrogenase 2	*MDH2*	CAACGTCCCTGTCATTGGTG	CAAGGGAGAAGACAAAGCGG
Pyruvate dehydrogenase α1	*PDHA1*	TGTGTGATGGTCAGGAAGCT	ACATGTGCATCGATCCTCCT
Glutamic-oxaloacetic transaminase 2	*GOT2*	AAGTAATGTCCCTGGTGCGA	CTCTGCCCTGTGTCACTACA
Glutaminase	*GLS*	GATTGCGAACGTCTGATCCC	TGCAACCTTTCCTCCAGACT
Glutamic-pyruvic transaminase 2	*GPT2*	TGCTGTGCTGTTTCCATCTC	TTTGGCAGGAATGAAGATCC
Glutamic-pyruvic transaminase	*GPT*	AAGGGCTGAGGGGTTAGGTA	AAGACCTGGGAAGACGGAGT
β-actin	*ACT-B*	ATAGCACAGCCTGGATAGCAA	AGGATTCCTATGTGGGCGAC

## Data Availability

Data will be made available upon request to Dr. Sachi Horibata (horibat2@msu.edu).
